# 

**Published:** 1888-02-11

**Authors:** 


### CONTENTS.

**The National Pension Fund Established** - - 327

**The National Pension Fund for Nurses.**—The Fund Incorporated.—What the Insurance World say - - - 328

**The Doctor's Pulpit.**—The Medicine of Work- - - 329

**Scotch Physicians and Surgeons.** By C. G. FURLEY.—Sir James Y. Simpson - - - - - - 330

**Medical Assurance Annuity Society** - - - 330

**Aids to Nursing.** By M. M. BASIL, M.A., M.B., Author of "The Commoner Accidents to Life and Limb" - - 331

**The Students' Column** - - - - - - 332

**The Practitioner's Clinic.** By A FAMILY PHYSICIAN.—Concerning Congestion - - - - - - 333

**Notes and News** - - - - - - - 334

**Everybody's Page** - - - - - - - 336

**Annotations.**—A Medical Philosopher.—A Scientific Preacher.—The Intellectual Woman of the Period - - - - 337

**Convalescent Homes in the Eastern Counties.** By OUR ROVING CORRESPONDENT - - - - - - 338

**Rachel Challice's Vow.** By LINA NEVILL, Author of "A Future on Trust," etc. - - - - - - 339

**The Editor's Letter-box.**—Oswestry Hospital Scandal, etc. 341

**Hints about Breakfast** - - - - - - 341

**The Nursing Mirror.**—A Laudable Ambition, etc. - - 341

**Questions and Answers.** - - - - - - 341

**Amusements with Profit for all Ages.**—For Men and Women.—Children's Corner.—Puzz'es, etc. - - - 342

The average issue of "The Hospital" now exceeds **10,000 COPIES WEEKLY,** and the circulation is steadily increasing

